# Comprehensive Assessments of RNA-seq by the SEQC Consortium: FDA-Led Efforts Advance Precision Medicine

**DOI:** 10.3390/pharmaceutics8010008

**Published:** 2016-03-15

**Authors:** Joshua Xu, Binsheng Gong, Leihong Wu, Shraddha Thakkar, Huixiao Hong, Weida Tong

**Affiliations:** Division of Bioinformatics and Biostatistics, National Center for Toxicological Research, U.S. Food and Drug Administration, 3900 NCTR Road, Jefferson, AR 72079, USA; zhihua.xu@fda.hhs.gov (J.X.); binsheng.gong@fda.hhs.gov (B.G.); Leihong.wu@fda.hhs.gov (L.W.); shraddha.thakkar@fda.hhs.gov (S.T.); Huixiao.hong@fda.hhs.gov (H.H.)

**Keywords:** genomics, RNA-seq, reproducibility, big data, next generation sequencing

## Abstract

Studies on gene expression in response to therapy have led to the discovery of pharmacogenomics biomarkers and advances in precision medicine. Whole transcriptome sequencing (RNA-seq) is an emerging tool for profiling gene expression and has received wide adoption in the biomedical research community. However, its value in regulatory decision making requires rigorous assessment and consensus between various stakeholders, including the research community, regulatory agencies, and industry. The FDA-led SEquencing Quality Control (SEQC) consortium has made considerable progress in this direction, and is the subject of this review. Specifically, three RNA-seq platforms (Illumina HiSeq, Life Technologies SOLiD, and Roche 454) were extensively evaluated at multiple sites to assess cross-site and cross-platform reproducibility. The results demonstrated that relative gene expression measurements were consistently comparable across labs and platforms, but not so for the measurement of absolute expression levels. As part of the quality evaluation several studies were included to evaluate the utility of RNA-seq in clinical settings and safety assessment. The neuroblastoma study profiled tumor samples from 498 pediatric neuroblastoma patients by both microarray and RNA-seq. RNA-seq offers more utilities than microarray in determining the transcriptomic characteristics of cancer. However, RNA-seq and microarray-based models were comparable in clinical endpoint prediction, even when including additional features unique to RNA-seq beyond gene expression. The toxicogenomics study compared microarray and RNA-seq profiles of the liver samples from rats exposed to 27 different chemicals representing multiple toxicity modes of action. Cross-platform concordance was dependent on chemical treatment and transcript abundance. Though both RNA-seq and microarray are suitable for developing gene expression based predictive models with comparable prediction performance, RNA-seq offers advantages over microarray in profiling genes with low expression. The rat BodyMap study provided a comprehensive rat transcriptomic body map by performing RNA-Seq on 320 samples from 11 organs in either sex of juvenile, adolescent, adult and aged Fischer 344 rats. Lastly, the transferability study demonstrated that signature genes of predictive models are reciprocally transferable between microarray and RNA-seq data for model development using a comprehensive approach with two large clinical data sets. This result suggests continued usefulness of legacy microarray data in the coming RNA-seq era. In conclusion, the SEQC project enhances our understanding of RNA-seq and provides valuable guidelines for RNA-seq based clinical application and safety evaluation to advance precision medicine.

## 1. Introduction

Pharmacogenomics studies evaluate how genes and genetic variations affect the individual response to therapeutic drug treatment [1]. Knowing whether a patient carries some specific genetic variations or has altered expression levels of some specific genes can help the physicians to individualize the drug therapy design, mitigate the chance for adverse drug events and to optimize the effectiveness of the drug and the dose. Such biomarkers also play an increasingly important role in drug discovery and development, from identifying actionable targets [2], drug repurposing [3], to drug safety assessment [4]. Pharmacogenomics is an integral and important part of precision medicine. In the past decade, microarray technology has enabled much of the advancement in pharmacogenomics as the primary technology for profiling gene expression. Recently, whole transcriptome sequencing, *i.e.*, RNA-seq, enabled by next generation sequencing, has provided a promising alternative to microarray for quantitatively studying gene expression [5]. RNA-seq also has many advantages, among them, a greater dynamic range, higher sensitivity towards low expression genes, alternative splicing detection, and gene fusion discovery [6]. However its reproducibility and utility in clinical settings are not clear. FDA-led Microarray Quality Control (MAQC) consortium launched the Sequencing Quality Control (SEQC) project to address these issues with the same data-sharing and consensus-pursuing model. With over 180 participants from about 70 organizations across industry, academia and government, the SEQC team labored over four years and completed the project by 2014. Data was archived and deposited in public repositories (GEO SuperSeries GSE47792). The present review provides a brief summary of some of the key findings of the SEQC project.

Several relatively independent but complementary components were included in the SEQC project: the core study with titration reference samples [7], neuroblastoma patient outcome prediction [8], comparison of RNA-seq with microarray through a comprehensive toxicogenomics data set [9], rat BodyMap project [10], and an investigation of biomarker transferability between RNA-seq and microarray [11]. Below, each component will be reviewed with an emphasis on data sets and key findings. However, this review is not comprehensive in covering all of the studies of SEQC project or related investigations of RNA-seq that were enabled by the SEQC samples and data sets.

## 2. Core Study with Titration Reference Samples

**Figure 1:**
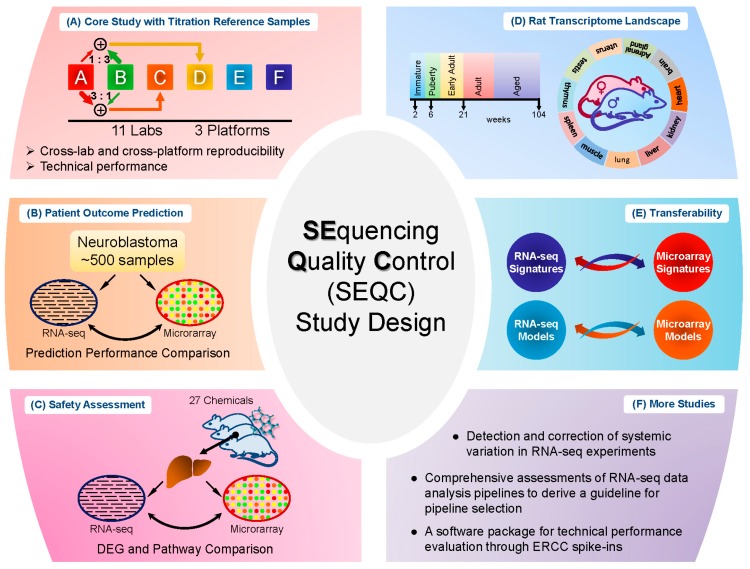
Overall study design for the SEQC project. (**A**) In the core study with titration reference samples, Samples A and B were augmented by Samples C and D in known mixing ratios 3:1 and 1:3, respectively. Synthetic RNAs from the External RNA Control Consortium (ERCC) were also added prior to mixing and sequenced separately (designated as Samples E and F). Samples were then distributed to multiple sites for library preparation and sequencing. The built-in truth through titration enabled assessment of cross-lab and cross-platform reproducibility and technical performance such as accuracy and precision; (**B**) The patient outcome prediction study used about 500 neuroblastoma samples to assess whether RNA-seq provides any advantage over microarray in predicting clinical outcomes. Various predictive models were constructed and compared between platforms; (**C**) The toxicogenomics study profiled the liver samples from over 100 rats treated with 27 chemicals representing seven toxicity modes of action. Gene expression data were generated by both RNA-seq and microarray platforms to compare their abilities to elucidate transcriptomic responses such as differentially expressed genes and pathways to toxicant treatments; (**D**) The rat transcriptomic BodyMap study aimed to provide a comprehensive survey of rat transcriptome landscape across sex, 11 organs, and four development stages; (**E**) The transferability study addressed the important question whether gene signatures and predictive models developed from microarray data can be directly applied to RNA-seq data and *vice versa*; (**F**) Aims/Results of additional SEQC studies.

As successfully applied in the MAQC-I project, titration study has proven to be a promising way to assess platform reproducibility and reliability [12]. Similarly, titration study was utilized here in the SEQC core study. Specifically, RNA samples from Universal Human Reference RNA and Human Brain Reference RNA were used as the starting material. Spike-in RNA mixes from External RNA Control Consortium (ERCC), as Samples E and F, were then added to the two reference RNA samples respectively to produce Samples A and B. Then Samples A and B were mixed with two fixed ratios, 3:1 and 1:3, to form Samples C and D [13]. In total, there are four built-in truths in the study design: (I) titration order, for instance, for a specific gene or transcript the measured expression level should be in the order A > C > D > B or inverse; (II) mixing ratios of Samples C and D were fixed, which could be used to test accuracy and reproducibility; (III) known concentration of transcripts in the spike-in ERCC mixes; and (IV) the spike-in RNAs were also titrated in Samples C and D. The samples were then distributed to multiple sites for sequencing. The design is depicted in Figure 1A. Several assessments were then performed based on these four built-in truths.

### 2.1. Gene Detection and Splice Junction Discovery

The influence of gene model, pipeline selection and sequencing platform was assessed in terms of gene detection and splice junction discovery [7]. For gene model assessment, by mapping 23.2 billion reads to three commonly-used gene models, NCBI AceView showed better performance than GENCODE and RefSeq. Aceview led to the highest percentages of mapped reads (97.1%, compared to 85.9% for RefSeq and 92.9% for GENCODE) and uniquely mapped reads even though its exons cover fewer bases than those in GENCODE. Additionally, with AceView, more known splice junctions were detected than the other two models under the same read-depth. Three different pipelines for RNA-seq mapping, NCBI Magic, r-make and Subread, were then tested for discovering novel juctions using AceView gene model. R-make found 50% more *de novo* junctions than Magic and Subread. In total, 2.6 million new splice junctions were discovered by at least one pipeline, however only 32% of them were called by all three methods. This relatively low concordance indicates that it is still challenging to reliably detect de novo splice junctions with current tools. For platform comparison, 97,117 unannotated junctions were discovered in the SOLiD data and 74,561 (86%) of them were concordant with those from HiSeq 2000 data.

Built-in truth was also applied to assess the accuracy of novel junctions to see whether their expression levels followed the titration order and the expected A/B mixing ratio (Truth I and II). Junctions with higher expression levels were usually concordant with the titration truth. Moreover, most of these unannotated junctions could be further validated by qPCR and even 18 of 22 junctions detected by only one pipeline could be qualitatively validated. As the last part of the assessment of gene detection and splice junction discovery, all sequencing data of Samples A–D from each site was pooled together and analyzed. In total, ~44,000 known genes, ~310,000 known exons and ~200,000 splice junctions were consistently detected across any pair of replicate sites, which constituted about 90%, 87%, and 83% of all known genes, exons and junctions.

### 2.2. Performance Assessment on Expression Analysis

For differential expression analysis, applying pipeline-dependent filters on *p*-value, fold change, and expression-level could significantly reduce the false positives while retaining high sensitivity [7,14]. By applying filters, up to 95% concordance in differentially expressed genes (DEGs) could be achieved among most pipelines, especially for those strongly expressed genes. These results imply the feasibility to conduct large cohort studies by incorporation of more samples sequenced at different sites or by different platforms.

Similarly, the built-in truth was applied to evaluate the performance of gene expression quantification. The majority of genes showed correct titration order (Truth I), where highly differentially expressed genes performed the best. Similarly, highly expressed genes performed better in mixing ratio recovery (Truth II and IV). However, the recovery ratio of spike-in controls showed a high variation across platforms and sites, indicating a clear batch effect on reproducibility. To facilitate the usage of the ERCC spike-in for technical performance evaluation of RNA-seq experiments, a software tool named “erccdashboard” [15] has been developed in the R statistical language and can be easily incorporated into other software analysis packages.

Additionally, gene expression measurements by different platforms, including TaqMan, Affymetrix Human Genome array, qPCR and RNA-seq platforms were compared. Relative expression levels showed a good agreement among different platforms but substantial variations were observed in absolute expression levels for numerous genes. This clearly demonstrated that relative expression measures were more reliable than absolute quantification. In summary, the core study with titration reference samples has demonstrated that RNA-seq is accurate and reproducible both across sites and across platforms. It has also confirmed the power of RNA-seq for discovering novel junctions. These findings have further consolidated the foundation of RNA-seq as a reliable and versatile tool for studying gene expression and thus will accelerate its application in pharmacogenomics and precision medicine.

## 3. Neuroblastoma Patient Outcome Prediction

Study of cancer is the disease area where most of the microarray and RNA-seq data are generated. The primary goal of the neuroblastoma patient outcome prediction component (see Figure 1B) was to systematically investigate the potential of RNA-seq based classifiers in predicting clinical endpoints in comparison to microarrays, especially for large cohort studies in cancer.

Gene expression profiles from 498 primary neuroblastoma samples were generated using both RNA-seq and microarray [8]. For gene identification, RNA-seq data was able to detect 48,415 AceView genes expressed above the background threshold, with 34,175 being coding genes and 14,240 non-coding genes. In contrast, microarray data only detected 21,101 AceView genes, indicating that RNA-seq could detect almost twice as many genes as microarrays. Thus RNA-seq was able to reveal more transcriptomic characteristics of the clinical samples.

To construct and assess predictive models, the whole cohort of 498 neuroblastoma samples was divided into the training and validation set. In total, 360 predictive models were constructed on six endpoints such as patient’s sex (SEX), event free survival (EFS_all), overall survival (OS_all), favorable disease outcome (FAV, a binary class label), and EFS and OS in high risk patients (EFS_HR and OS_HR). These six endpoints were designed to cover a broad range of predictive difficulties, from easy (SEX, FAV) and medium (EFS_all and OS_all) to difficult (EFS_HR and OS_HR). Multiple data analysis teams were then involved in the model construction with various data processing pipelines and expression features for RNA-seq. As a result, the predictive performance was largely determined by the endpoints and neither the technology platform (RNA-seq *vs.* microarrays) nor the data processing pipeline of RNA-seq could significantly affect the model prediction performance. Additionally, the correlation of performance between internal and external validation for microarray- and RNA-seq-based models was almost identical.

In summary, RNA-seq was demonstrated to offer more utilities than microarrays in determining the transcriptomic characteristics of cancer. However, the more extensive transcriptomic information provided by RNA-seq would not improve the model prediction performance, as RNA-seq and microarray-based models performed similarly in clinical endpoint prediction. This large scale RNA-seq study with clinical samples established the utilities of RNA-seq in clinical settings for further development of precision medicine.

## 4. Safety Assessment through Toxicogenomics Study

In addition to cancer study, toxicogenomics is also a critically important area for transcriptome analysis. Different from clinical studies, the chemical treatment effect could be well controlled and the aim of toxicogenomics study in SEQC was to address the question whether RNA-seq would be more suitable than microarrays for elucidating and predicting toxicity mechanism of various chemical treatments [9]. For this study, 27 chemical compounds were selected to represent seven distinct toxicity modes of action; and, for each compound, three biological replicates and matched controls were profiled by both microarray and RNA-seq [16]. The samples were then divided into the training and test set for evaluation of model development to predict the mode of action. This study design (see Figure 1C) enabled a set of comparison between microarray and RNA-seq. The result showed that the treatment effect could lead to different degrees of concordance between the two platforms (RNA-seq and microarray) in terms of DEG detection, whereas compounds with a marked treatment effect showed more consistency between platforms in DEGs and pathways than compounds with a weak treatment effect. Additionally, most of the discrepancy between the two platforms was caused by lowly-expressed genes, where highly-expressed genes were more consistent between the two platforms. Furthermore, the discrepancy was due to the low sensitivity of microarray in measuring lowly-expressed genes, according to the comparison of both platforms to qPCR data. As for model performance comparison, the overall predictive accuracy was similar for both platforms (61% for RNA-seq and 58% for microarray).

In brief, the toxicogenomics component showed that if the objective is only to build genomic markers-based predictive models, either platform was suitable for use. However, RNA-seq would perform better on profiling lowly-expressed genes which could reveal the treatment effect more precisely and comprehensively, especially for weakly effected groups, and also could discover more specific features such as noncoding RNAs, splice variants and novel junctions. Thus RNA-seq offers additional potential in safety assessments.

## 5. Rat Transcriptomic Body Map

The rat has been extensively used as a model organism for scientific studies, and the main objective of the Rat Body Map component (see Figure 1D) was to comprehensively survey its transcriptome across multiple organs and developmental stages [10]. In detail, 320 Fischer 344 rat samples from 11 different organs were collected and profiled by RNA-seq, generating about ~13.2 billion 50 bp sequence reads [17]. Rat AceView transcriptome was adopted as the gene model for data processing with UCSC rat genome rn4 as the reference genome. In total, 40,064 genes, 65,167 transcripts, 31,909 alternatively spliced transcript variants and 2367 non-coding genes/non-coding RNAs were found based on AceView gene model. As a result, only few thousand of genes were observed as commonly expressed across all organs and at all developmental stages, where most of genes showed organ-specific differential expression across the lifespan. In particular, an overview landscape of organ-dependent, development-dependent and sex-specific expression was investigated. This Rat Body Map is expected to provide a comprehensive platform for biomedical researchers by enabling improved assessments of drug efficacy and toxicity with the rat model, which helps advance better understanding of human disease and treatment strategies.

## 6. Gene Signature Transferability between RNA-seq and Microarray

RNA-seq is poised to replace microarray and ushers in the era of RNA-seq for transcriptome profiling. Large volumes of data, predictive models and biomarkers have accumulated as a result of the wide application of microarray in biomedical research. This raises an important question: Can predictive models and biomarkers developed from microarray data be directly applied to RNA-seq data and vice versa to leverage the enormous investments in microarray? The gene signature transferability investigation [11] addressed this question by conducting a comprehensive evaluation of the transferability of predictive models and signature genes between microarray and RNA-seq using two large clinical data sets with one of them being the neuroblastoma data set generated by the SEQC consortium.

The investigation began with an examination of the complexity of cross-platform sequence correspondence in the analysis of three human and two rat data sets. Three levels of mapping complexity were identified and then used to group the genes. To account for different modeling complexity, k-nearest neighbors (k-NN), nearest shrunken centroids (NSC), and support vector machine (SVM) were adopted to represent three levels of complexity from the simplest to the most complex. In total, 240,096 predictive models were examined. Across all combinations of mapping and modeling complexities, signature genes derived from one platform can be transferred to build models for the other platform with little loss in prediction performance. However applying predictive models directly from one platform to another would lead to mixed results. For most combinations of mapping and modeling complexities, microarray-based models could accurately predict RNA-seq-profiled samples. On the other hand, RNA-seq-based models were less accurate in predicting microarray-profiled samples and were affected both by the choice of modeling algorithm and the gene mapping complexity. This investigation suggests continued usefulness of legacy microarray data and established microarray biomarkers and predictive models in the forthcoming RNA-seq era.

In addition, the SEQC consortium also made a coordinated effort to address the critical challenges in choosing RNA-seq analysis pipelines to achieve improved gene expression quantification and downstream predictive modeling of disease outcome. A comprehensive investigation of 278 representative RNA-seq data analysis pipelines consisting of 13 sequence mapping, three quantification, and seven normalization methods was conducted to assess their impact on gene expression accuracy and precision; sensitivity in detecting low-expression genes; specificity in detecting DEGs; and downstream prediction of disease outcome. Our results reveal that RNA-seq pipelines that produced better gene expression usually resulted in better prediction models. A guideline was then provided for the selection of reliable RNA-seq data analysis pipelines. The manuscript [18] is currently under revision at Nature Methods.

## 7. Perspective

With this brief review, we aim to summarize some of the key SEQC studies and major findings to spur interest and more thorough examination of these studies and the data sets. To facilitate further research in RNA-seq assessment and development of analysis methods, the comprehensive data sets, including sequencing data and related microarray and qPCR data, have been documented thoroughly and deposited into public data repositories. As a result, the SEQC data sets have already been utilized for studying data normalization methods [19], detecting and correcting systemic variation [20], and uncovering gene regulation in response to toxicant exposure [21]. It is worthy to emphasize that the RNA samples used in SEQC studies were extracted from cell line cultures (reference samples), fresh frozen tissues (rat liver samples), or snap-frozen tissues (neuroblastoma patient samples). The high quality of these samples may have contributed to the high consistency observed between RNA-seq and microarray. It would be interesting to revisit this comparison with some degraded RNA samples, e.g., those extracted from formalin fixed paraffin embedded (FFPE) samples. As the SEQC consortium derived consensus about RNA-seq continues to gain more acceptance in the research community, it is our hope that the findings and recommendation guidelines from the consortium would help accelerate the discovery and development in biomedical research, particularly in pharmacogenomics, in advancing precision medicine.
